# Beyond Competency: Developing Critical Digital Capabilities in Nursing Students Through Freirean Pedagogy

**DOI:** 10.1111/nin.70011

**Published:** 2025-03-12

**Authors:** Matthew Oliver Wynn

**Affiliations:** ^1^ School of Nursing and Advanced Practice Liverpool John Moores University Liverpool England

**Keywords:** education, nurse education, social theory

## Abstract

The digitalisation of healthcare is transforming nursing practice, presenting unique opportunities and challenges that demand more than technical competence from nursing professionals. Despite the growing integration of digital tools, nursing remains in the ‘foothills of digital transformation’, with significant gaps in the critical and theoretical frameworks required to navigate this shift effectively. This article explores how Paulo Freire's critical pedagogy may address these gaps by fostering critical digital skills in nursing students. Drawing on Freire's concepts of problem‐posing education, conscientization, dialogue and praxis, the article proposes a pedagogical model that encourages students to critically examine the socio‐political and ethical implications of digital tools within their practice. By aligning Freirean principles with contemporary nursing challenges, the article argues for a shift away from solely competency‐based frameworks toward educational approaches that promote reflective, dialogical, and ethically informed engagement with technology. The limitations of Freirean pedagogy, including its difficulty in evidencing direct outcomes, are discussed alongside its potential to cultivate a philosophically engaged nursing workforce capable of navigating the complexities of a digital healthcare environment and its associated impact on the profession. This approach underscores the importance of preserving nursing's core ethical and relational values while embracing the transformative potential of digital technologies.

## 1 Introduction

Digitalisation is a pressing concern in contemporary nursing, as technology increasingly shapes both the delivery and nature of care. The rapid integration of digital tools is set to fundamentally transform healthcare environments, indicating that nurses not only adopt new skills but also potentially reconsider their professional roles and responsibilities. Despite this, in the UK, a recent independent report on the state of the National Health Service (NHS) reported that it remains ‘in the foothills of digital transformation’ and that despite the opportunities presented by digital technologies, they have ‘not radically reshaped services*’* and that data from digital systems are largely untapped for research (Darzi 2024, 103). With nursing being the largest profession in healthcare, it is arguable that the influence exerted by its leadership, culture and practices may have a disproportionate influence on the digitalisation of healthcare services. A recent scoping review on developing digital competencies in nursing professionals, however, reported that there remains little in the way of well‐evidenced and structured approaches to this internationally (Tischendorf, Hasseler, et al. 2024). The authors called for further *theoretically* based frameworks and modules as the basis of curricula in degree programs.

However, establishing such theoretical approaches to digital health education for nurses presents its own challenges. Traditional nursing theories, often rooted in human‐centred and practice‐oriented perspectives, have struggled to capture the evolving complexities of nursing within the context of digital healthcare (Wynn et al. 2023). This contrasts with professions such as medicine, where the knowledge and competency requirements for effective practice can be relatively easily identified in relation to their impacts on the diagnosis and treatment of pathology, respectively, as per the less ambiguous ‘medical model’. As technologies like electronic health records, telehealth, artificial intelligence and robotics permeate healthcare settings, the ambiguity surrounding nursing's knowledge base arguably leaves it particularly vulnerable to external technological influences that may not align with the values and expectations of nurses themselves. This is illustrated in the context of robotic technologies. Where nurses may perceive human touch and ‘humanness’ as essential components of nursing (Pepito et al. 2023); a recent study evaluating the acceptability of robots by patients report that in circumstances where human care is lacking, robots are likely to be accepted, particularly among men (Hertog et al. 2024). This illustrates a potential misalignment between nurses' professional values and patient expectations in technologically mediated care. While nurses may view their role as fundamentally human‐centred, emphasising empathy, presence, and touch, patients, particularly in situations where human care is inadequate or unavailable, may demonstrate a more pragmatic approach, prioritising access to assistance over the nature of the provider.

This tension underscores the vulnerability of nursing to external technological impositions. If healthcare systems prioritise efficiency and cost‐effectiveness, robotic solutions may be introduced not as supplementary aids but as replacements for human care, despite nurses' concerns about the erosion of the relational aspects of their profession. Without a clearly defined epistemological foundation that articulates what aspects of nursing are nonnegotiable, the profession risks being shaped by external technological agendas rather than by the values and expertise of nurses themselves.

Furthermore, this dynamic raises ethical and practical questions about the delegation of care tasks to nonhuman entities. If patient acceptance of robotic care is largely contingent on the absence of human alternatives, as suggested by Hertog et al. (2024), then its widespread implementation could risk normalising reduced human involvement in care, further shifting expectations and potentially redefining what ‘good’ nursing looks like. This highlights the need for nursing to assert its own technological agency, to critically engage with, rather than passively adopt, digital innovations, ensuring that technological integration enhances rather than diminishes the core values of nursing practice.

This article argues that Paulo Freire (1970) philosophy of critical pedagogy offers a valuable framework for addressing these vulnerabilities. Freire's concept of *problem‐posing education*, which advocates for a learning environment that challenges students to question and critically engage with knowledge, contrasts sharply with the ‘banking model’ of education, where information is passively received by students. In the nursing profession, this banking model may lead to uncritical adoption of digital technologies, positioning them as ends in themselves rather than tools to enhance the profession's humanistic and ethical commitments. While many nursing curricula incorporate active learning and emphasise critical analysis, there is limited evidence to suggest that this extends specifically to critical engagement with digital technology in nursing education. Tischendorf, Hasseler, et al. (2024) highlight that digital competence training is often ad hoc rather than systematically integrated, and that nursing pedagogy lacks a clearly defined framework for developing critical approaches to digital technology. This gap suggests that without structured critical engagement, there is a risk of passive acceptance of digital tools rather than their thoughtful, ethical integration into nursing practice.

Freire's concept of *conscientization*, or the development of critical consciousness, encourages nurses to examine the role of digital tools within a framework that considers professional identity, the socio‐political and historical context of the nursing profession. The relevance of Freire's critical pedagogy in nursing education is further supported by Wynn and Garwood‐Cross's (2024) application of Actor‐Network Theory (ANT), a social theory developed by Callon (1984), Law (1992) and Latour (2005). This application of ANT frames nursing not as a traditional profession with a clear definable knowledge base and practices, but instead as a complex assemblage of human and nonhuman actors, each influencing the profession's practices. These actors may include technologies, policies, nursing theories and models, education curricula and governments in addition to individual nurses themselves. By viewing nursing through this sociological lens, ANT highlights the role that digital technologies and institutional policies play alongside human actors in shaping nursing practice. Without a clear theoretical foundation, nursing is at risk of having these technological and external policy influences dominate its trajectory, potentially leading to confusion around the knowledge nurses need in an increasingly digitalised healthcare environment. Freire's approach provides a critical counterbalance, providing an educational model that empowers nurses to engage with digital technologies thoughtfully and purposefully, in alignment with the profession's core ethical and relational commitments.

## 2 Nursing's Epistemological and Theoretical Ambiguity

Nursing has long grappled with questions about its definition and professional identity, with multiple frameworks attempting to articulate its scope. Unlike other health professions that have a singular, widely accepted definition, nursing remains fluid, reflecting its evolving and context‐dependent nature. This fluidity is tacitly acknowledged by the International Council of Nurses (ICN), which has historically provided multiple definitions of nursing and is currently (as of 2025) engaged in a project to revise and update its definition. While this ongoing refinement demonstrates the profession's adaptability, it also highlights a struggle to coalesce around a singular, universally accepted framework. Without a clear, stable definition, nursing's identity remains open to interpretation, which has implications for policy, education, and professional boundaries. This ambiguity, while reflective of nursing's dynamic and multifaceted role in healthcare, also leaves the profession vulnerable to external forces that shape its trajectory, particularly as new technologies and healthcare models emerge.

Beyond the challenge of defining the profession, nursing's epistemological foundation remains complex and contested. Traditional nursing theories, such as Watson (1997) theory of human caring, Orem (1986) self‐care deficit theory, and Leininger (1978) transcultural theory, have provided important conceptual frameworks that emphasise empathetic, patient‐centred care and the social dimensions of healing. While these theories have significantly influenced nursing education and practice, they do not fully encompass the profession's expanding scope, particularly in the face of digital transformations in healthcare. Recent theoretical work has sought to address this gap. For example, a scoping review by Wynn et al. (2023) identified eight nursing theories that engage with digital technologies, focusing on issues such as how technology facilitates care, the necessity of technological competence, and the ethical implications of digital tools. Their analysis identified three overarching themes: technology as an agent within the care environment, the necessity of technological competence, and the role of digital tools in enabling nurses to ‘know’ their patients. These contributions challenge the notion that nursing lacks theoretical engagement with digital transformation. However, without a stable epistemological foundation, the profession's knowledge base is often shaped by external forces.

One consequence of nursing's epistemological instability is its frequent subordination to other professional knowledge systems, particularly the medical model. This is evident in the development of advanced nursing practice roles, where ‘nursing knowledge’ is often framed through the lens of biomedical expertise rather than as an independent epistemological domain. Wynn and Garwood‐Cross (2024) argue that this positioning is not necessarily a reflection of nursing theory itself, but rather a response to economic and resource pressures within healthcare systems, driving role expansion to meet workforce demands. The cost‐effectiveness comparisons between advanced nurse practitioners and doctors (Abraham et al. 2019) suggest a lack of meaningful differentiation between the two professions in terms of knowledge or skill, reinforcing the notion that nursing's epistemological foundation remains unstable. Abraham et al. (2019) explicitly acknowledge this dynamic, stating: ‘Physician‐centric models cannot effectively meet the demand for patient care, and primary care providers from other disciplines, such as advanced practice nurses, are needed to optimize chronic disease care delivery’. They further note that: *“*Thirty‐nine countries have developed policies to shift primary care tasks from physicians to APNs to meet the demand for patient care’. Similar narratives were presented by Martin‐Misener et al. (2015) who state that ‘nurse practitioners provide similar services to those for whom they are substituting, usually physicians’ and that ‘Usually the health service aim of the former is to reduce cost or workload or to address workforce shortages’. This discourse clearly illustrates that the development of nursing roles is, in some cases, driven by workforce shortages and medical resource constraints, rather than a clear articulation of distinct nursing knowledge or theoretical contributions justifying changes in nurses' practice. Crucially, this observation is not intended to denigrate the practice of nurses in advanced roles. Both studies cited here report the efficacy and safety of these roles. This observation merely serves to illustrate the external influences on the nature of nursing practice and its associated knowledge requirements.

In the absence of a unified theoretical foundation, competency‐based, and generic approaches to digital skill development have become prominent. For example, the National Health Services 2017 ‘Health and Care Digital Capabilities Framework’, which notably does not mention nursing and has since been superseded by profession‐specific frameworks. Or more generic digital capabilities frameworks such as that provided by Jisc (2024) which are necessarily atheoretic with regards to the specifics of nursing practice. These approaches typically focus on specific, measurable outcomes, aiming to ensure that nurses acquire a set of skills deemed necessary, often by non‐nurses, for working in digital healthcare environments. However, competency‐based frameworks, while practical in a technical sense, may fall short in equipping nurses with a critical understanding of digital tools within the broader context of nursing practice. Instead, they risk producing a workforce skilled in technology but lacking the reflective and critical perspectives needed to navigate complex digital environments thoughtfully. This may result in what has been described as ‘e‐iatrogensis’ or harm related to the use of health information technology (Weiner et al. 2007). When digital competencies are taught as isolated technical skills, the implications of these tools on patient care and professional identity are often neglected. In addition, the technical nature of new technologies such as artificial intelligence, pose entirely unique ethical challenges (Morley and Floridi 2024) which defy neat competency‐based training approaches. For example, the opacity of many AI algorithms, often referred to as the ‘black box’ phenomenon, means that the logic underlying these tools is not always transparent to end‐users, including nurses (Hassija et al. 2023). This lack of transparency creates a fundamental tension in nursing practice, as nurses may be expected to trust and rely on AI systems without fully understanding how they work or how they arrive at certain recommendations. This may erode the autonomy of nurses and compromise their role as patient advocates, if they feel compelled to defer to AI recommendations that they cannot interrogate critically.

The emphasis on competency in digital skills often reflects both broader healthcare trends that prioritise efficiency, standardisation, and accountability; and the established culture of competency‐based approaches in broader nurse education. These approaches have been the subject of robust critique more generally due to, among other things, their arguably criticality‐killing nature (Collier‐Sewell et al. 2023). While the objectives of competency‐based approaches to education are laudable, their application to nursing may be at odds with the profession's inherently relational and individualised approach to care. The absence of a stable epistemological foundation means the profession lacks a strong, unified theoretical basis from which to critically examine the role and purpose of digital technology in patient care, or to determine what exactly should be included in structured competency‐based frameworks should these persist. This issue is highlighted in one study exploring pre‐registration students' perspectives on digital technology which reported that students experienced a form of identity crisis due to the capabilities of contemporary technologies (Wong et al. 2023). This highlighted a critical disconnection between the perceptions of student nurses about what it meant to be a nurse, and the capabilities of modern digital technologies. Thereby illustrating the unique paradigmatic challenges associated with nurse education in a rapidly digitalising world.

## 3 Developing Critical Consciousness in Nursing Education

Paulo Freire's critical pedagogy and his concepts of problem‐posing education, conscientization, dialogue and praxis, provides nursing educators with a framework to equip students to critically engage with digital technologies rather than passively accepting their roles in healthcare. Freire's model challenges traditional hierarchies where educators are mere dispensers of knowledge and students passive recipients, a setup that may undermine students' development of autonomy and critical thought (Freire 1970). The potential value of Freirean approaches to nursing pedagogy have been proposed historically to address issues associated with a broader conception of nursing as an ‘oppressed’ group, this is argued to be a consequence of the oppression of women more generally (Harden 1996). This is of obvious significance in a profession which remains to be dominated by women globally. Notably, gender itself has also been shown to be a potentially significant factor influencing the use of technology by nurses in a narrative review of technology use and acceptance by Wynn et al. (2023). More recently, Freirean philosophy has been applied to address perceived political issues in nursing, including that of nursings' ‘colonial practices of whiteness’, which is considered an oppressive force by some contemporary nurse education scholars (Iheduru‐Anderson and Waite 2024), and for addressing relational violence between nurses (Pitcher and Browne 2023). These examples highlight the association of Freirean pedagogy with addressing the structural challenges faced by the nursing profession beyond the technicalities of hands‐on care. In a Freirean framework, problem‐posing education repositions digital tools from mere technical competencies to components of a broader healthcare network that must be critically examined. This approach emphasises teaching students not the mechanics of these tools but encourages them to question their purpose, ethical implications, and the impacts on patient care and professional identity. The key differences between competency‐based approaches and Freirean approaches can be seen in Table 1. It is worth noting that within competency‐based pedagogic approaches, reflection is in some cases included. However, it is argued that tokenistic inclusions of reflective activities within what are primarily competency focussed approaches to pedagogy is dissimilar in nature and potential outcomes to a focussed application of Freirean principles to address more abstract and less measurable pedagogical outcomes among nurses, for example critical consciousness of digital technology and its impacts on nurse identity.

**Table 1:** Competency‐based vs. Freirean approaches.

Attribute	Competency‐Based Framework	Freirean Pedagogy
**Focus**	Technical proficiency	Critical consciousness
**Learning approach**	Instructor‐led, predefined tasks	Dialogue, problem‐solving
**Evaluation methods**	Standardised tests, checklists	Reflective journals, group discussions
**Outcomes**	Skill acquisition, task completion	Ethical engagement, critical thinking
**Philosophical Basis**	Efficiency and standardisation	Empowerment and *transformation*

To illustrate potential approaches to implementing Freirerean approaches in digital nurse education, each key principle consczientization, problem‐posing, dialogue, and praxis will be considered.

## 4 Conscientization

*Conscientization*, as defined by Freire (1970), is the process of developing critical awareness of one's social reality through reflection and action, enabling individuals to perceive and challenge the structures that shape their experiences. This critical awareness enables nurses to discern the underlying motives behind digital innovations often introduced under banners like ‘efficiency’ and ‘accountability’. Freire's principles also question the limits of competency‐based approaches to digital education. Instead of positioning competencies as final achievements, a Freirean model frames them as entry points into deeper reflection and dialogue. Nursing students, for example, may learn the operation of specific digital tools but are also encouraged to examine their effects on clinical judgement, patient autonomy, and the nurse–patient relationship.

In the context of nursing education, conscientization can empower students to critically examine the implications of digital technologies in their professional practice. The most direct approach to developing critical consciousness, taking into account the recommendations of Wong et al. (2023) to engage with the impacts of digital technology on nursing identity, would be to explore the question ‘what is a nurse?’. Despite this potentially seeming obvious question, the ongoing definitional work by the ICN suggests this is perhaps a mistake. There is even debate currently around the name given to the body which represents ‘nursing knowledge’ with some scholars called for, what has been described as ‘superficial and shallow’, attempt to rename it ‘nursology’ (Parse 2019). Encouraging nurses to actively engage with this question of what it means to be a nurse, and what it is nurses do (or should) know, is perhaps the first step in unlocking the critical and creative attitudes identified as essential by van Houwelingen et al. (2024) to increase engagement of nurses with technological developments.

Other examples of activities which may yield conscientization include analysing case studies involving ethical dilemmas related to AI diagnostic tools or robotic assistants. This offers a structured way for students to reflect on the impact of these technologies on patient care, autonomy, and ethical dimensions. These discussions, conducted in small groups, may encourage nurses to move beyond surface‐level engagement, recognising the socio‐political and ethical forces driving technological adoption. Group projects mapping the influence of technologies like electronic health records (EHRs) (such as the potential for e‐iatrogenesis) on nursing decision‐making and patient relations may further deepen this critical awareness, helping students see both the opportunities and unintended consequences of technology.

## 5 Problem‐Posing

Freire's concept of *problem‐posing education* shifts the educational model from a passive transfer of knowledge to an active process where students and educators cocreate knowledge through questioning and dialogue, fostering critical thinking and transformative learning. ‘The role of the problem‐posing educator is to create, together with the students, the conditions under which knowledge at the level of the doxa is superseded by true knowledge, at the level of the logos*’* Freire (1970, 54). In nursing education, this approach can be implemented through real‐world problem scenarios that encourage students to grapple with the complexities of digital healthcare, ideally drawing on the students' pre‐existing understanding of digital technologies. For example, students might explore dilemmas such as balancing the time required for EHR documentation with providing quality patient care or addressing the ethical challenges of algorithmic biases in AI systems. These collaborative exercises not only enhance problem‐solving skills but also help students recognise how technology influences care quality, patient outcomes, and professional practice. Similarly, exercises involving dummy EHR datasets may enable students to formulate questions that address nursing care issues while critiquing how and why digital data is underutilised, as highlighted by Darzi (2024). This approach fosters a mindset where students are not mere users of technology but active participants who can question, challenge and innovate within digitised healthcare environments.

## 6 Dialogue

Dialogue, as Freire (1970) emphasises, is a mutual process of learning where knowledge is co‐constructed through reflective, respectful exchanges that acknowledge and value diverse perspectives. Dialogue plays a fundamental role in developing critical consciousness according to Freire. In dialogical classrooms, students contribute diverse perspectives, learning from each other's insights and questioning assumptions, which helps them assess the influence of digital technology on nursing care. By engaging in these dialogues, students begin to recognise that technology in healthcare is rarely value‐neutral; its design, deployment and impact often reflect broader economic, political and social forces.

Nursing education can integrate dialogical learning by fostering debates and discussions that encourage critical thinking and shared exploration of digital technologies. Ideally, dialogue should be focussed around ‘generative themes’ (Freire 1970). These themes represent words, ideas or images of central relevance to the lived experience of the students. Freire recommends that as part of research to develop new programs of education educators should

select not only the words most weighted with existential meaning (and thus the greatest emotional content), but also typical sayings, as well as words and expressions linked to the experience of groups in which the researcher participates. These reveal longings, frustrations, disbeliefs, hopes and an impetus to participate.

(Freire 2021, 46).

These words, sayings and expressions should form the basis for the dialogue. Sources of such generative themes may be derived from the growing body of qualitative literature exploring the perceptions of nurses towards the integration of digital technology into practice. For example, an integrative review on the perceptions of nurses towards artificial intelligence in nursing practice by Lora and Foran (2024) identified several key issues nurses were concerned about. These included ‘deskilling’ a lack of ‘human touch’, ‘discrimination’ to name a few. These themes can be used to facilitate targeted dialogue in education settings. For example, discussion around questions such as ‘will technology de‐skill nurses?’, ‘is digital technology compatible with the concept of *care*?’ *‘*what should the role of human carers in a healthcare system with robots?’ may help students explore diverse viewpoints and critically assess how such tools impact the nurse–patient relationship, professional autonomy, and the nature of care. Socratic discussion may further deepen this exploration by addressing questions like ‘What is lost when AI influences care decisions?’ or ‘How does technology shape the nurse's role and identity?’ These discussions, grounded in the real‐world concerns and anxieties of nurses rather than focussed on more technical issues related to technology, encourage students to consider the ethical and relational dimensions of technology, questioning whether digital tools enhance or undermine core nursing values. These discussions themselves may identify further themes from which dialogue may develop.

## 7 Praxis

Praxis refers to the cyclical process of reflection and action aimed at transforming one's reality through critical engagement and meaningful intervention. This capacity for ongoing reflection is essential in a dynamic profession like nursing, where tools and practices rapidly change. Encouraging students to critically assess technology throughout their careers helps develop practitioners skilled in digital tools yet steadfast in advocating for patient‐centred applications of technology, resisting applications that are purely efficiency‐driven or dehumanising. Crucially, Freire advocates for programs which would ‘develop the impatience and vivacity which characterize search and invention’ (Freire 2021, 41). This is of clear significance given the regular calls by nurse leaders internationally to involve nurses more in the development of technologies. A recent study exploring the phenomenon of limited nurse‐input with technology development identified that attitudes including motivation, creativity and criticality were key to facilitating this involvement (van Houwelingen et al. 2024). Without sufficient critical engagement with the paradigmatic issues facing nursing, it is perhaps unsurprising that cultures may develop in nursing which facilitate apathy towards engagement with new opportunities presented by digital technologies.

In nursing education, praxis can be nurtured through activities that blend reflective learning with actionable insights. Reflective journaling during clinical placements, for instance, would allow students to document their interactions with digital tools, analyse how these tools shape patient care and professional roles, and critically assess their impact. Sharing these reflections in group settings promotes collective learning, enabling students to refine their perspectives and propose improvements. Role‐playing exercises may further reinforce praxis by immersing students in scenarios where they simulate the use of digital tools, such as telehealth consultations, and reflect on the effects of these tools on communication, trust, and the relational aspects of nursing. Lastly, designing or improving nursing technologies as part of a project encourages students to apply their theoretical knowledge to real‐world challenges. This may draw on contemporary theories, such as ‘technological creativity as caring theory’ developed by Bahari et al. (2021); which emphasises the nurses' non‐technical role in the creation of new nursing technologies. Instead, drawing on the nurse's creativity and ‘innovator characteristics’ to engage others (including non‐nurses) in projects which result in the development or improvement of technologies (Bahari et al. 2021). This process is proposed to be an expression of ‘caring’ itself. These activities connect technological innovation with nursing's core values of care and compassion. By integrating reflection and action, praxis‐oriented education ensures that nursing students develop not only technical proficiency but also the critical and ethical capabilities necessary to shape the future of digital healthcare and adapt their nursing philosophy to the new digital context.

Through application of Freirean pedagogy, nursing students may be encouraged to view digital tools as part of a larger network of human and nonhuman actors, aligning with the Actor‐Network Theory perspective of nursing as described by Wynn and Garwood‐Cross (2024). However, the goal remains to help students assert their agency (via changes in praxis) within this network, becoming active subjects rather than passive objects within the larger assemblage of nursing. Without this sense of agency and awareness, nursing students may find themselves uncritically adopting technologies.

## 8 Limitations of Freirean Approaches to Developing Digital Capabilities and Implications for Practice

While a Freirean approach offers significant benefits for fostering critical engagement with digital technologies, it is not without its limitations. One of the main challenges is the difficulty in evidencing the direct impacts of this approach. Unlike competency‐based frameworks, which can be assessed through measurable outcomes such as technical proficiency, Freirean pedagogy focuses on the development of critical consciousness and reflective thinking, qualities that are inherently harder (if not impossible) to quantify. The lack of concrete metrics for evaluating its effectiveness may pose challenges for educators and institutions seeking to justify its integration into curricula. To address this, educators could develop qualitative evaluation methods, such as reflective journals, focus groups, and narrative case studies, to capture evidence of critical thinking and ethical engagement. Additionally, combining Freirean pedagogy with competency‐based metrics in hybrid evaluation models could help bridge this gap, demonstrating both technical proficiency and critical consciousness. Freirean approaches cannot replace the need for technical skills training. While it encourages students to critically assess and reflect on digital tools, such reflection must be complemented by robust, hands‐on education that ensures nurses are competent in using these tools effectively. A Freirean approach is best viewed as an enhancement to technical training rather than a substitute, providing the critical perspective necessary to contextualise and question the role of technology in practice. A counterpoint to this, however, is that a robust technical training without the establishment of a clear philosophy associating the value of engagement with technology with ‘good’ nursing practice may result in nurses who are technically capable on specific technologies, but otherwise disengaged from the broader project of exploiting technology optimally for the benefit of patients.

There is evidence to suggest that nurse educators currently lack confidence and expertise in digital technology for health (Zhao et al. 2024). There is also evidence that there is a lack of consensus surrounding what should constitute an appropriate education in digital health for nurses (Zhao et al. 2024, Tischendorf, Heitmann‐Möller, et al. 2024). These issues highlight two key issues, firstly that traditional competency‐based education processes are unlikely to succeed with the current knowledge‐gap in digital health among nurse educators. Secondly, that the underlying theoretical gaps underpinning nursing itself may render ambiguous the necessary content of a nursing education in digital health. It could also be argued that the pace of technological development itself, for example in the case of AI, make creation and maintenance of standardised competencies challenging, even within professions with well‐defined epistemology and ontology. In both cases, a criticalist Friearean approach may be of value by allowing opportunity to bridge this impasse via co‐construction of knowledge via dialogue.

There is a potential risk of relativism in Freirean pedagogy. While co‐constructing knowledge through dialogue is valuable, it may inadvertently lead to the perception that all perspectives are equally valid. This can create tension with evidence‐based practice, where clear standards and guidelines are essential. Nursing educators must carefully balance open‐ended dialogue with the need to reinforce evidence‐based care principles. Additionally, the focus on critical reflection could risk creating a workforce that is highly critical but lacks actionable problem‐solving strategies. For instance, students may become disillusioned with technological adoption without developing the skills to advocate for or design better systems. Pairing reflective exercises with practical problem‐solving activities and broader leadership skills may help mitigate this risk. To address these risks, educators should establish boundaries for dialogue by grounding discussions in evidence‐based practice and using structured frameworks to evaluate ideas. Pairing reflective activities with solution‐oriented projects, such as designing mock digital tools or proposing enhancements to existing systems, can help students translate critical awareness into practical skills and strategies.

Finally, the implementation of Freirean pedagogy implies educators adopt a more neutral stance, prioritising the development of independent perspectives among students rather than advocating for specific technologies or approaches. This could lead to outcomes where students critically reject certain digital tools or technologies, a position that may conflict with institutional or professional goals to promote digitalisation. For educators who are also champions of technological adoption, this neutrality can be particularly challenging, as it necessitates creating space for critical dissent rather than promoting specific technological solutions to nursing problems in practice. Chambers (2019) argues, however, that complete neutrality in dialogic pedagogy is not only not feasible but also undesirable. He instead argues for a ‘compatibilist’ interpretation of Freirean pedagogy, suggesting that directive teaching and dialogue are not mutually exclusive but can coexist in a balanced educational approach. Educators must embrace their role as guides, using directive methods judiciously to create structured opportunities for students to critically engage with complex issues. Chambers (2019) advises educators to foster environments where students feel empowered to challenge assumptions while being equipped with the tools to analyse and propose solutions constructively. This involves, perhaps paradoxically, openly sharing the educator's own perspectives as starting points for dialogue, thereby removing any potential for the perception of neutrality. In addition, educators should model virtues such as humility, respect, and critical curiosity, and emphasising the ethical implications of digital technologies. A balanced educational environment is necessary where critical dissent is encouraged, but students are also taught how to evaluate digital tools constructively and propose alternatives. In the context of digital technologies in nursing, this could, for example, utilise the wide range of published evaluation frameworks for digital health technologies (Krick 2021). Inevitably, this requires both skill and intent on behalf of nurse educators to ensure that education claiming to utilise Freirean pedagogy does not become a veiled effort to convince students or nurses of a particular perspective on digital technology and its significance in nursing (intentionally or otherwise). Continuous reflection by educators would be necessary to prevent this in addition to the active avoidance of making normative claims on epistemologically controversial issues. This might include, for example, perspectives on the role of robot technology in elderly care, which is subject to conflicting views between patients and professionals (Wynn 2024, Hertog et al. 2024).

Despite these challenges, Freirean pedagogy has the potential to cultivate a more philosophically engaged nursing workforce. By interrogating the paradigmatic and socio‐political influences on nursing practice, students may develop a deeper understanding of how these forces shape their profession and its relationship with technology. This critical awareness may support the digitalisation of nursing practice more effectively by equipping nurses to advocate for technologies that align with the profession's ethical and relational commitments. Nurses who appreciate the socio‐political and paradigmatic impacts of digital tools are arguably better positioned to navigate the complexities of implementation, ensuring that digitalisation serves both patient care and the profession's values.

Despite considerable focus on the philosophy of Freire within the health education literature, there remains a dearth of empirical evidence indicating the value of implementing learning interventions based on his ideas. Ideally, nurse educators using this approach should seek to build this evidence base to determine what, if any, the value of this form of education may be, and what impacts it may have on students of nursing. Methods suited to this may include qualitative studies analysing reflective journals, focus groups or long‐term follow‐up of students to establish evidence of their engagement with digital technology in practice.

## 9 Conclusion

The integration of digital technologies into nursing practice presents both unprecedented opportunities and challenges, demanding a thoughtful approach that prioritises critical engagement over mere technical proficiency. Nursing's theoretical and epistemological ambiguities make it especially vulnerable to external technological pressures that may not align with the profession's humanistic and ethical commitments. Freire's critical pedagogy offers a powerful framework for developing a reflective and resilient nursing workforce, one capable of navigating the complexities of digital tools with a patient‐centred focus. By fostering critical consciousness through problem‐posing education, dialogical learning based on pertinent generative themes related to digital technology in nursing, and structured reflection on praxis, nursing education may equip students not only to develop digital competencies but to critically evaluate the broader impacts of these tools on their professional identity, ethical responsibilities, and patient relationships. As nursing continues to evolve within an increasingly digitalised healthcare landscape, embracing Freirean principles may support the profession in preserving its values while engaging thoughtfully with technological advancements. Ideally, nurse educators seeking to implement these principles should seek to gather empirical evidence of its impact on students.

## Ethics Statement

The author has nothing to report.

## Conflicts of Interest

The author declares no conflicts of interest.

## Data Availability

The author has nothing to report.
